# Study on Flexural Behavior of Corroded I-Shaped Steel Beams Strengthened with Hybrid CFRP/GFRP Sheets

**DOI:** 10.3390/ma16145080

**Published:** 2023-07-19

**Authors:** Yiming Han, Nan Li, Guangxi Zhang

**Affiliations:** 1 College of Resources, Shandong University of Science and Technology, Tai’an 271019, China; hz4707979@163.com; 2 Shandong Geotechnical Engineering Big Data Intelligent Analysis Technology Characteristic Laboratory, Tai’an 271019, China; 3 School of Architectural and Engineering, Weifang University of Science and Technology, Shouguang 262700, China; sueiyuxinghan@163.com

**Keywords:** corroded defective steel beam, CFRP/GFRP, bending resistance, reinforce

## Abstract

How to effectively reinforce steel structures with rust and damage is an important research topic in the engineering field. This article takes the rusted I-beam as the research object and analyzes the effects of different CFRP/GFRP reinforcement methods on the bending performance of rusted I-beams through experimental research with a total of 1 unreinforced beam and 7 CFRP/GFRP-reinforced beams. The results show that when the number of CFRP/GFRP-reinforced layers is the same, replacing some of the CFRP with GFRP and using different interlayer mixed laying sequences have little effect on the bending bearing capacity of rusted I-beams. The number of CFRP/GFRP-reinforced layers is the key factor affecting the bending bearing capacity. At the same time, numerical simulation was conducted using finite element software to study the stress distribution and stress development law of the reinforced beam, and the numerical simulation results were consistent with the experimental results.

## 1. Introduction

During the use of steel structure buildings, corrosion damage is difficult to avoid. The annual economic losses caused by steel corrosion in the United States reach up to 27.6 billion US dollars [1], while the annual economic losses caused by steel corrosion in China can also reach 3% to 5% of GDP [2]. In the past 20 years, FRP has been proven to be a successful repair method for external reinforcement of structures, with advantages such as high strength, lightweight, corrosion resistance, and degradation resistance [3,4,5,6,7].

In the application of using FRP to reinforce steel structures, the high cost of using CFRP alone for reinforcement hinders the development of this technology. Therefore, GFRP with lower prices but similar material properties has received attention. To combine the advantages of the two, researchers proposed the concept of using two or more continuous fiber materials to enhance composite materials with the same resin matrix. Through the hybrid reinforcement test of CFRP/GFRP fabric [8,9], it was found that the addition of GFRP cloth can enhance the crack resistance and ductility performance of concrete components. The addition or replacement of CFRP with GFRP fabric reduces the cost of reinforcement, but the reinforcement effect does not show a significant decline. 

Lin Zhang et al. [10] found that both CFRP fabric and GFRP fabric can improve the bearing capacity of steel beams. Xiaobing He et al. [11] studied the interlayer hybrid effect of CFRP/GFRP fabric using ultimate tensile tests, demonstrating that externally bonded CFRP/GFRP fabric can significantly enhance the crack resistance and bearing capacity of beams. Attaria et al. [12] found that the strength of reinforced concrete beams strengthened with double-layer CFRP/GFRP sheets increased by 114% and exhibited good ductility. Dejun Mao [13] showed that the cracking load and ultimate bearing capacity of CFRP-cloth-reinforced beams are greater than those of GFRP-cloth-reinforced beams. The crack distribution, crack development morphology, and specimen failure form of the two types of reinforced beams are similar, and the ductility performance of GFRP-cloth-reinforced beams is better. Xiaodong Zhang et al. [14] conducted experiments on the reinforcement of beams and columns with Intrinsically Hybrid Fiber Reinforced Polymer (IHFRP), proving that the plastic deformation capacity and energy absorption capacity of beams strengthened with IHFRP sheets have significantly improved, improving the bearing capacity, stiffness, and ductility of the beams. Zongcai Deng et al. [15] demonstrated that using carbon/aramid/glass interlayer hybrid fiber cloth reinforcement can effectively improve the bending performance of corroded beams. Wei et al. [16] studied the performance of CFRP cloth, GFRP cloth, and other fiber layer hybrid bonding to reinforce reinforced concrete beams. Research has found that the higher the ultimate tensile strength and distribution of fiber materials, the more significant the improvement in the ultimate bearing capacity of the test beam. 

Based on the above research, the hybrid reinforcement of steel beams with CFRP/GFRP sheets can effectively improve their bending performance and have good reinforcement effects on defective steel beams. Compared with CFRP sheet reinforcement, the hybrid reinforcement effect of CFRP/GFRP sheet is similar and the cost is reduced, which improves the economic benefits of FRP sheet reinforcement technology.

At the same time, domestic and foreign scholars mostly simulate the damage of corroded steel beams through the mechanical cutting of the steel beam web and flange [17,18]. Compared to the damage of steel beams under natural corrosion, it is difficult to accurately reflect the bending performance of steel beams after corrosion damage.

In response to the shortcomings in current research, the bending behavior of corroded I-steel beams strengthened with hybrid CFRP/GFRP sheets is studied by means of a bending test in this paper. The main factors, such as the number of reinforcement layers and laying order, were analyzed, and numerical simulations were conducted with finite element software.

## 2. Materials and Methods

### 2.1. Test Piece Processing

**Figure 1:**
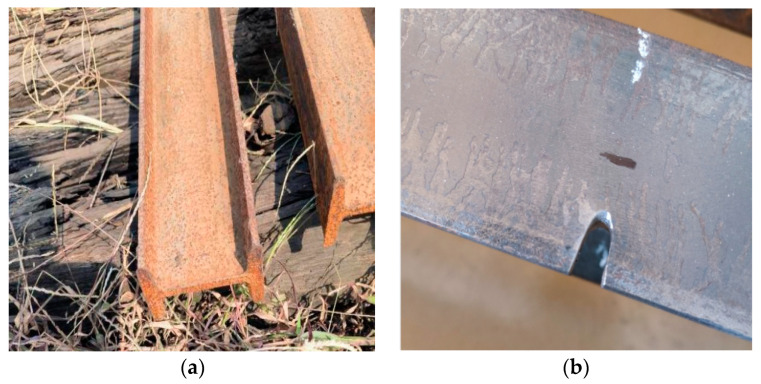
(**a**) Steel beams stored in the open air; (**b**) Rust processing of steel beams.

In this experiment, I8 I-shaped steel beams were stored in the open air and naturally corroded for 6 months. The corrosion status of the steel beams is shown in Figure 1a. Simulate corroded steel beams by mechanically machining the lower flange of the beam span. Cut the lower flange of the steel beam at the mid-span position of the specimen by 15 mm, accounting for 60% of the width of the single-side lower flange. The steel beam cut is shown in Figure 1b.

Paste CFRP/GFRP cloth along the length direction of the steel beam to reinforce the steel beam, with a length of 950 mm.

**Figure 2:**
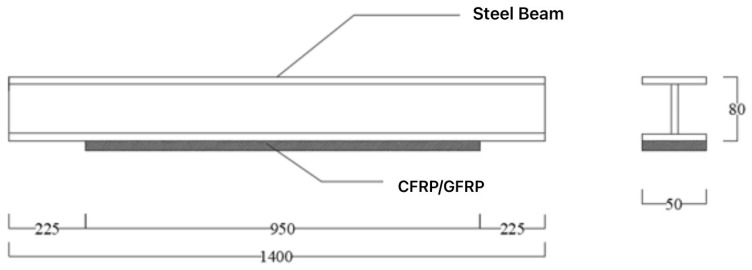
Design drawing of test piece.

At the ends of the fabric, CFRP fabric U-clamps with a width of 50 mm are used for anchoring. The length of the steel beam is 1400 mm, the calculated span is 1200 mm, and the size of the steel beam is 80 × 50 × 5 × 6 (mm), the dimensions of the test steel beam are shown in Figure 2.

The experimental design includes 1 unreinforced beam and 7 reinforced beams. All steel beams have undergone natural corrosion for 6 months, and the lower flange of the steel beam span has been mechanically processed. The interlayer mixed layer sequence is from the inner layer to the outer layer. As shown in Table 1.

### 2.2. Material Properties

The steel beam adopts Q235B I-beam, with a yield strength of 245 MPa and a tensile strength of 393 MPa. The steel beam is weighed using a platform scale before and after rust damage. The comparison of the quality of the steel beam after rust damage is shown in Table 2.

The CFRP cloth uses CAIDINGKE brand 300 g first-class standard cloth, and GFRP cloth uses Chongqing Canyue New Material Co., Ltd. HS2 cloth (Chongqing, China). The material characteristics of CFRP fabric, GFRP fabric, and adhesive are provided in the testing report provided by the manufacturer. The main mechanical properties of the material are listed in Table 3 and Table 4.

When the steel beam is stored in the open air for 6 months, precipitation and moisture in the air will react with the corrosion of the steel beam. The surface of the steel beam will form rust layers of different rust depths. At the same time, due to mechanical entry and material processing in the open air, other impurities such as dust, oil, rust, etc. will accumulate on the steel beam. Before the test, rust removal treatment is carried out on the steel surface [19], and the surface of the steel beam is polished with an industrial angle grinder to remove the rust and impurities in order to keep the bonding surface of the steel beam clean.

**Table 4:** Performance parameters of fiber cloth impregnated rubber material.

Testing Items	Testing Basis	Detection Result
Tensile Strength	National Standard [20]	56.2 MPa
Tensile Modulus of Elasticity		2932 MPa
Elongation		2.33%
Bending Strength		89.5 MPa, and there is no fragmented damage
Compressive Strength		81.0 MPa

### 2.3. Loading Device

**Figure 3:**
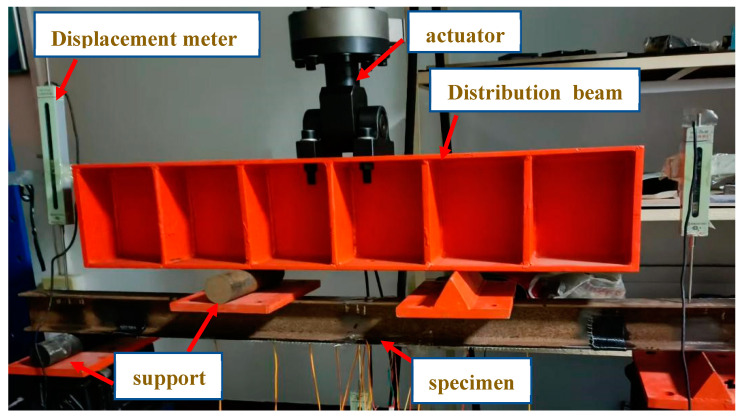
Schematic diagram of four-point bending loading device.

The loading device for this experiment is shown in Figure 3. The specimen bears a symmetrical concentrated load during the experiment, with a load action spacing of 500 mm and a loading point of 100 mm from the support. In the early loading stage, the experimental phenomenon of the components was not significant, so the design loading increased by 6 kN per stage. When the structure approached the yield load, the loading load decreased to 3 kN per stage, and the loading speed should be slow and uniform throughout the entire process. From the beginning of loading to the end of loading, it should be controlled at 3 min, and after loading is completed, the load should be held for 10 min before continuing to load. The location of the strain gauge is shown in Figure 3.

## 3. Test Results

### 3.1. Failure Model

**Figure 4:**
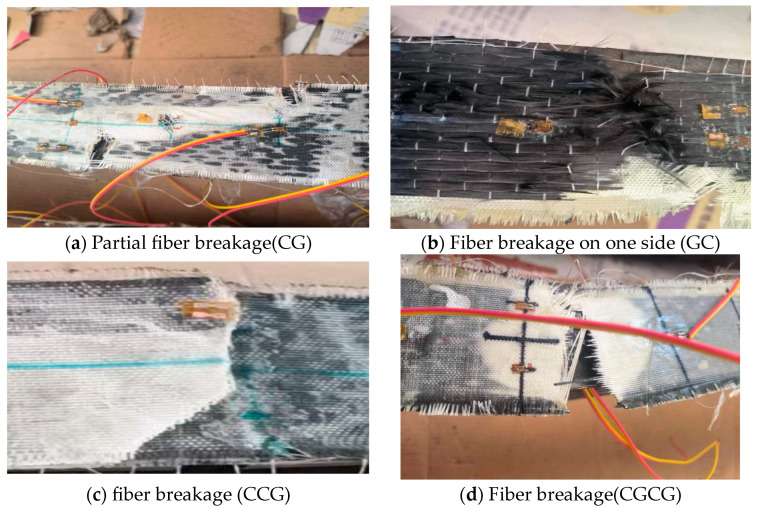
Details of specimen failure.

Based on the different deformation modes during the failure of 8 test beams, 2 failure modes were identified, namely the bending torsional instability failure of the middle section of the steel beam and the fracture and detachment of the CFRP/GFRP cloth from the steel beam. The failure modes of each beam are shown in Table 5, and the detailed failure of the specimen is shown in Figure 4.

The failure mode of bending torsional instability of the middle section of the steel beam is due to the lack of structures to ensure the local stability of the members and transfer the concentrated force at the place where the support and concentrated load are applied, resulting in the failure of force transmission during the loading process, which reduces the stability and torsional resistance of the steel beam. When the steel beam is subjected to the upper load, it begins to generate bending moments in the main plane of maximum stiffness. Due to the significant difference in the inertia moments of the two main axes of the I-beam section, both in-plane and out-of-plane deformations will occur, manifested as downward bending of the steel beam and lateral displacement accompanied by twisting deformation. As the steel beam is not designed with stiffeners, the torsional stiffness and bending stiffness outside the bending moment action plane of the steel beam is not large, and there is not enough lateral support to prevent it from generating lateral displacement and torsion. As the load value continues to increase, the steel beam ultimately experiences bending and torsional instability failure outside the bending moment action plane.

In the failure mode of CFRP/GFRP fabric breaking and detaching from the steel beam, the first manifestation is partial fiber breakage of CFRP/GFRP fabric, accompanied by colloid detachment. This indicates that the stress distribution of CFRP/GFRP fabric is very uneven during the loading process. The detachment of the adhesive layer reflects that the bonding force between the steel beam, colloid, and CFRP/GFRP cloth is insufficient to ensure that the CFRP/GFRP cloth and the steel beam remain in a cohesive state throughout the pressure-bearing process. Due to the bending deformation of the steel beam, the colloid and CFRP/GFRP cloth are forced to change accordingly. However, the elastic modulus between the colloid and CFRP/GFRP cloth is different, resulting in inconsistent deformation between the various parts during the increase in load and mid-span displacement, causing the CFRP/GFRP cloth to break and detach from the steel beam.

Analyzing the failure modes of CFRP/GFRP fabric, it can be divided into two types of failure modes. One type of fiber fracture is perpendicular to the direction of force, and the other is the first step of fiber detachment or fracture at the edge. As the load increases, oblique development fracture occurs along the direction of fiber force.

This is determined by the anisotropic material characteristics of CFRP/GFRP cloth. When the fiber bundle of CFRP/GFRP fabric is parallel to the tensile direction, it has excellent tensile strength. However, once the fiber bundle is tilted or even perpendicular to the line of force, the tensile strength significantly decreases. Further, because the reinforcement of CFRP/GFRP fabric is manually bonded, the thickness and distribution of the adhesive layer are difficult to fully control, which is also one of the reasons why CFRP/GFRP fabric cannot fully exert its performance.

### 3.2. The Influence of GFRP Fabric Reinforcement Layers

**Figure 5:**
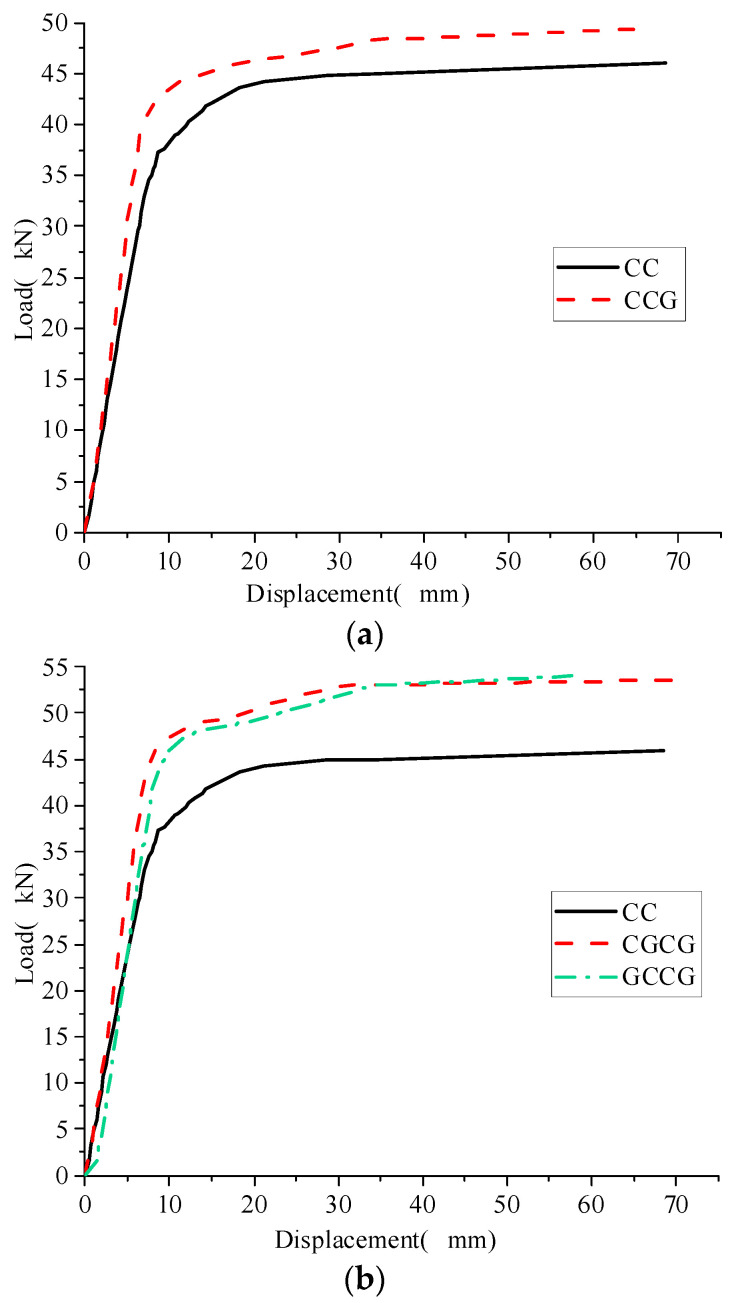
Comparison of load-displacement curves of specimens with different GFRP cloth reinforcement layers. (**a**) load-displacement curves of CC and CCG; (**b**) load-displacement curves of CC, CGCG and GCCG.

We designed control specimens with the same number of CFRP reinforcement layers and different GFRP reinforcement layers to study the improvement of GFRP reinforcement layers on the bending performance of corroded I-shaped steel beams. Compare and analyze the load-displacement curves of CC, CCG, CGCG, and GCCG, as shown in Figure 5.

It can be seen from Figure 5. The addition of GFRP cloth did not change the stress deformation law of the reinforced beam, and the load-displacement curve of the reinforced beam showed the same development trend. It can be seen from the comparison of the curves in Figure 5a. Under the same amount of CFRP reinforcement, the yield load of CCG is 40.78 kN, and the ultimate load is 48.39 kN, which is 9.27% and 5.26% higher than CC, respectively. From the comparison of the curves in Figure 5b, it is found that the yield loads of CGCG and GCCG are 43.64 kN and 42.95 kN, which are increased by 16.93% and 15.09%, respectively, compared to CC. The ultimate loads are 53.60 kN and 54.12 kN, which are increased by 16.60% and 17.73%, respectively, compared to CC. The number of reinforcement layers of GFRP fabric is directly proportional to the increase in yield load and ultimate load of the reinforced beam. GFRP fabric effectively suppresses the plastic development of the tensile zone of the reinforced beam and enhances the bending bearing capacity of the corroded I-shaped steel beam. The experimental data are shown in Table 6.

### 3.3. The Influence of Interlayer Mixed Layering Order

**Figure 6:**
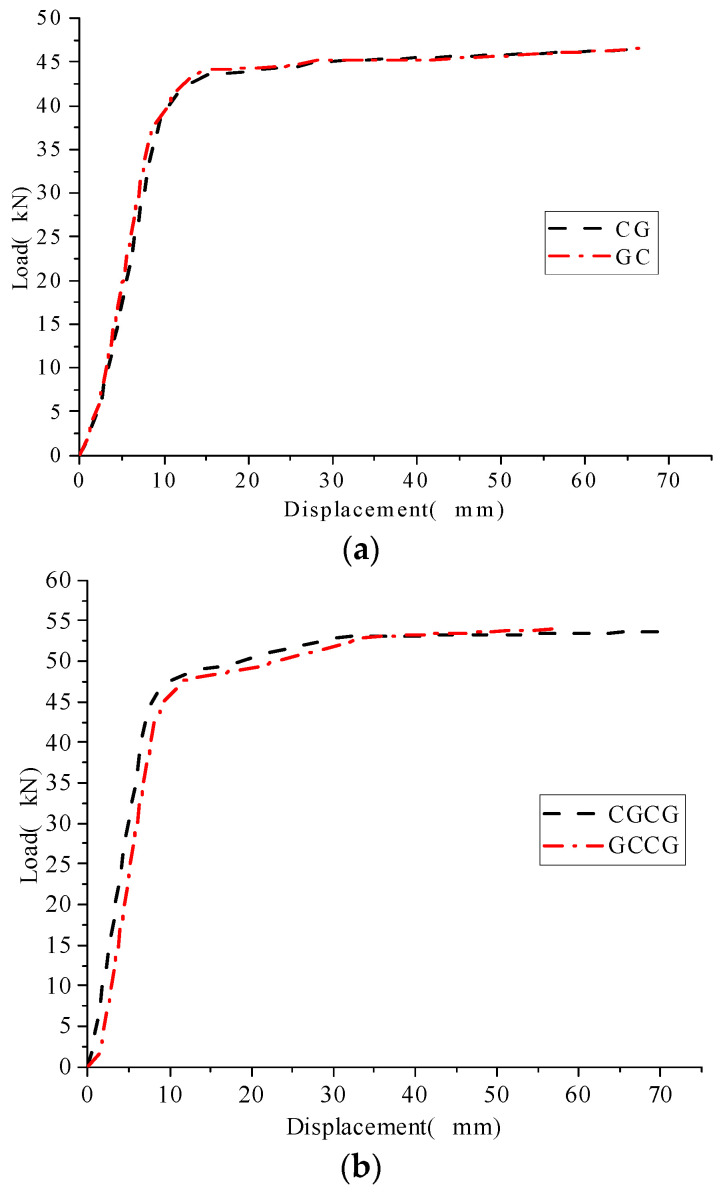
Comparison of load-bearing capacity of specimens with different interlaminar mixed layer sequences. (**a**) load-displacement curves of CG and GC; (**b**) load-displacement curves of CGCG and GCCG.

Compare and analyze the load-displacement curves of CG, GC, CGCG, and GCCG, as shown in Figure 6. It can be seen from Figure 6. The addition of GFRP cloth did not change the stress deformation law of the reinforced beam, and the load-displacement curve of the reinforced beam basically coincided. It is found from the comparison of the curves in Figure 6a. Compared to GC, the yield load of CG decreased by 1.07% and the ultimate load increased by 1.06%. It is found from the comparison of the curves in Figure 6b. Compared with GCCG, the yield load of CGCG decreased by 1.58% and the ultimate load increased by 0.97%. Prove that the mixed layer sequence between GFRP/GFRP layers has little effect on the bearing capacity of corroded I-shaped steel beams. The experimental data are shown in Table 7.

### 3.4. Comparison of Reinforcement Effects of GFRP Fabric Partially Replacing CFRP Fabric

**Figure 7:**
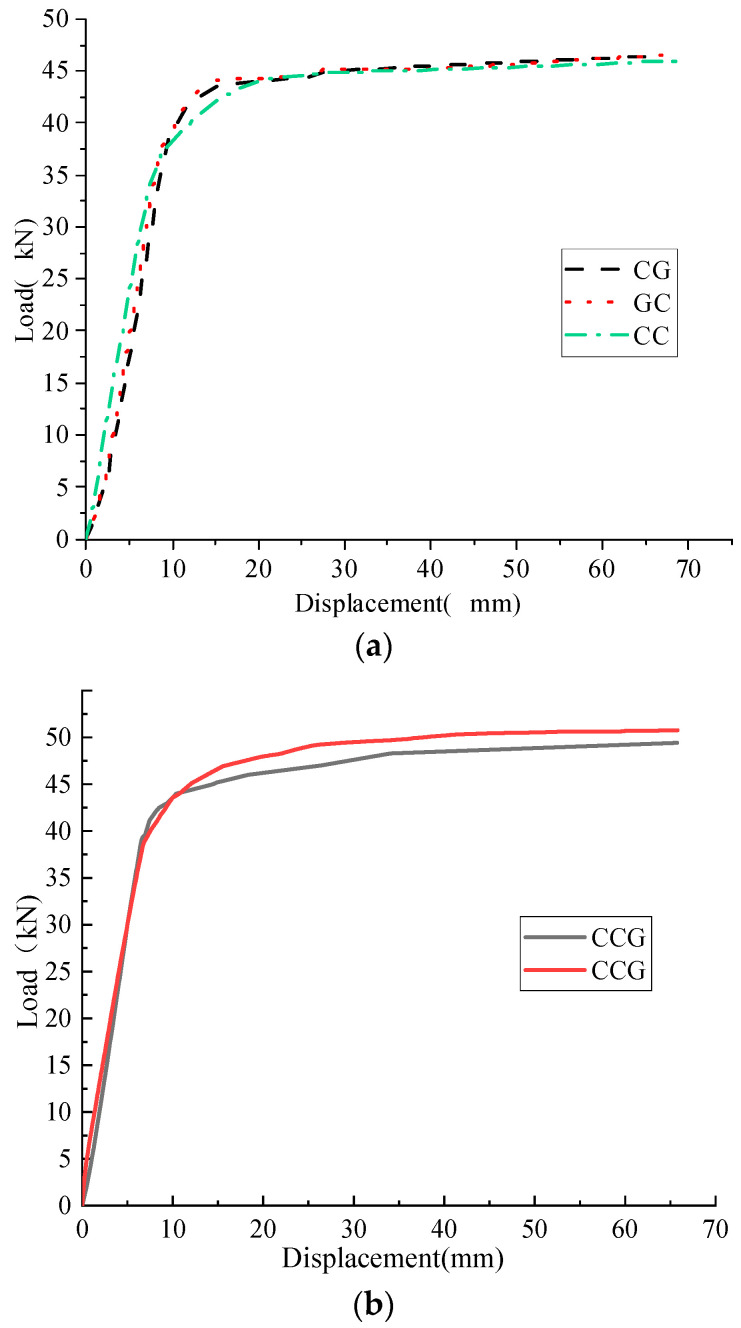
Comparison of load-displacement curves of specimens reinforced with GFRP fabric instead of CFRP. (**a**) load-displacement curves of CG, GC and CC; (**b**) load-displacement curves of CGG and CCG.

Compare and analyze the load-displacement curves of CG, GC, CGCG, and GCCG, as shown in Figure 7. The addition of GFRP cloth did not change the stress deformation law of the reinforced beam, and the load-displacement curve of the reinforced beam basically coincided. From the curve comparison in Figure 7a, it is found that the yield load of CG is increased by 3.08% compared to CC, and the ultimate load is increased by 1.07%. Compared with CC, GC increased the yield load by 1.98% and the ultimate load by 1.33%. It is found from the comparison of the curves in Figure 7b. Compared with CGG, the yield load of CCG increased by 1.13% and the ultimate load increased by 2.13%. It has been proven that replacing CFRP fabric with GFRP fabric has a good reinforcement effect, and the impact on the bearing capacity of corroded I-shaped steel beams is minimal. The comparison of experimental data is shown in Table 7.

### 3.5. Comprehensive Analysis of the Experiment

**Figure 8:**
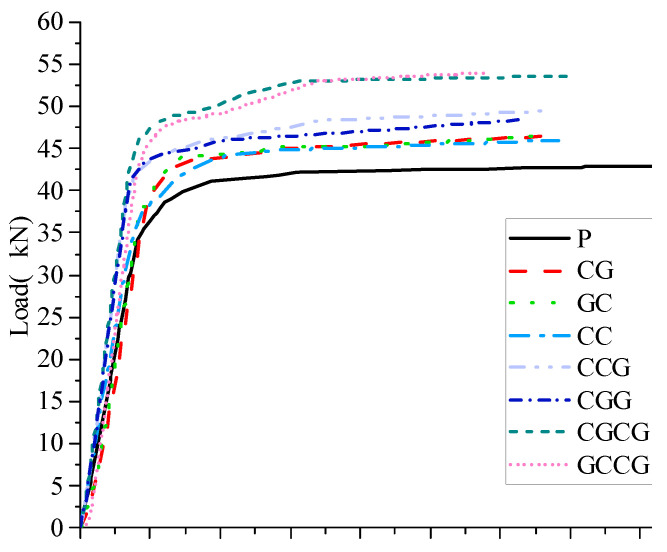
Load-displacement curve comparison.

Analyze the stiffness of the reinforced beam based on the load-displacement curves obtained from 8 specimen beams. From the comparison of load-displacement curves in Figure 8, it can be seen that after being reinforced with CFRP/GFRP sheets, the reinforced beams show a decrease in mid-span displacement.

Compared with unreinforced beams, the yield load increase rate of the beams strengthened with 2 layers of CFRP/GFRP fabric is between 2.78% and 5.95%, and the ultimate load increase rate is between 7.23% and 8.65%. The yield load increase rate of the 3 layers of CFRP/GFRP fabric is between 12.31% and 13.58%, and the ultimate load increase rate is between 12.88% and 15.28%. The yield load increase rate of the 4 layers of CFRP/GFRP fabric is between 17.68% and 20.19%, and the ultimate load increase rate is between 25.03% and 27.34%. It can be seen that the number of reinforcement layers of CFRP/GFRP fabric is the most important factor affecting the reinforcement effect, and the increase rate of yield load after reinforcement is lower than the increase rate of ultimate load. The experimental data are shown in Table 8.

## 4. Numerical Simulation of Mixed Reinforcement of Corroded I-Shaped Steel Beams with CFRP/GFRP Sheets

### 4.1. Model Building

The steel beam is simulated using C3D8R solid units, and the regional seed arrangement is 10 mm per unit. Both CFRP fabric and GFRP fabric are modeled using shell elements (S4R). The CFRP/GFRP fabric adopts structured grid division units, and the regional seed layout adopts 10 mm per unit. The adhesive layer element adopts a three-dimensional eight-node cohesive force element (COH3D8), which can be used to simulate the bonding and sliding of the contact interface. The adhesive layer is divided into elements using the sweep method, with the thickness direction designated as the separation direction. The CFRP/GFRP fabric adopts structured grid division units, and the regional seed layout adopts 20 mm per unit.

In the grid division of the model, it is necessary to use as few grid units as possible to save computer resources and speed up the calculation time. Under different element sizes, the number of elements increases significantly, but the stress peaks obtained are approximately equal. Considering the balance between CPU time and finite element calculation accuracy, 20 mm is selected as the regional seed layout for the numerical model in this paper.

The ideal elastic-plastic model is selected as the constitutive model for subsequent finite element analysis. Steel has obvious elastic and yield stages, but when the stress reaches the yield point, the steel strain can reach 2% to 3%. Although there is no damage to such large deformation, the structure or component is no longer suitable for continuing to bear the load, so the elastic-plastic stage is ignored and the steel is simplified as an ideal elastic-plastic material.

In the simulation, the support and load loading area is simplified as rigid pads with equal contact surfaces. Since the pads have always been in the elastic stage, the elastic modulus of the pads is set to 100 times that of the steel, and the yield strength is not defined.

GFRP and CFRP exhibit various anisotropy as composite materials. The modulus and stress of hybrid materials comply with linear mixing laws, proving that CFRP and GFRP conform to the principle of equal strain during the stress process of hybrid materials [21,22,23,24], and the stress-strain relationship is linear [25,26,27,28,29,30]. The material parameters are set according to the testing report in Section 2.2 of this article. To define the elastic stage, it is necessary to select Engineering Constants in the model material elasticity to input the elastic moduli E1, E2, and E3 of the material in three main directions, as well as the Poisson’s ratios Nu12, Nu13, Nu23, and shear moduli G12, G13, and G23 in planes 12, 13, and 23.

Coupling the upper surface of the cushion blocks used to apply load on the upper part of the I-shaped steel beam to the center reference points of their respective upper surfaces, directly applying force to the coupling points. Tie connections are used between the I-beam and the cushion block, between the I-beam and CFRP or GFRP, and between CFRP and GFRP. This model simulates a four-point bending test in the form of a simply supported beam. Therefore, the support is constrained in the form of a simply supported beam, with one end constraining the translational degrees of freedom in the X and Y directions, and the other end constraining the translational degrees of freedom in the Y direction.

Use the Explicit analysis step to calculate the entire process of steel beam bending. Use the History Output mode to output reaction force (RF2) and displacement (U2).

Compare the load-displacement curve obtained from simulation and experiment, and calculate the deviation value by taking the load value at the mid-span displacement of 50 mm. The simulation and experiment are basically consistent. The deviation values are all less than 4%.

### 4.2. Stress Nephogram

**Figure 9:**
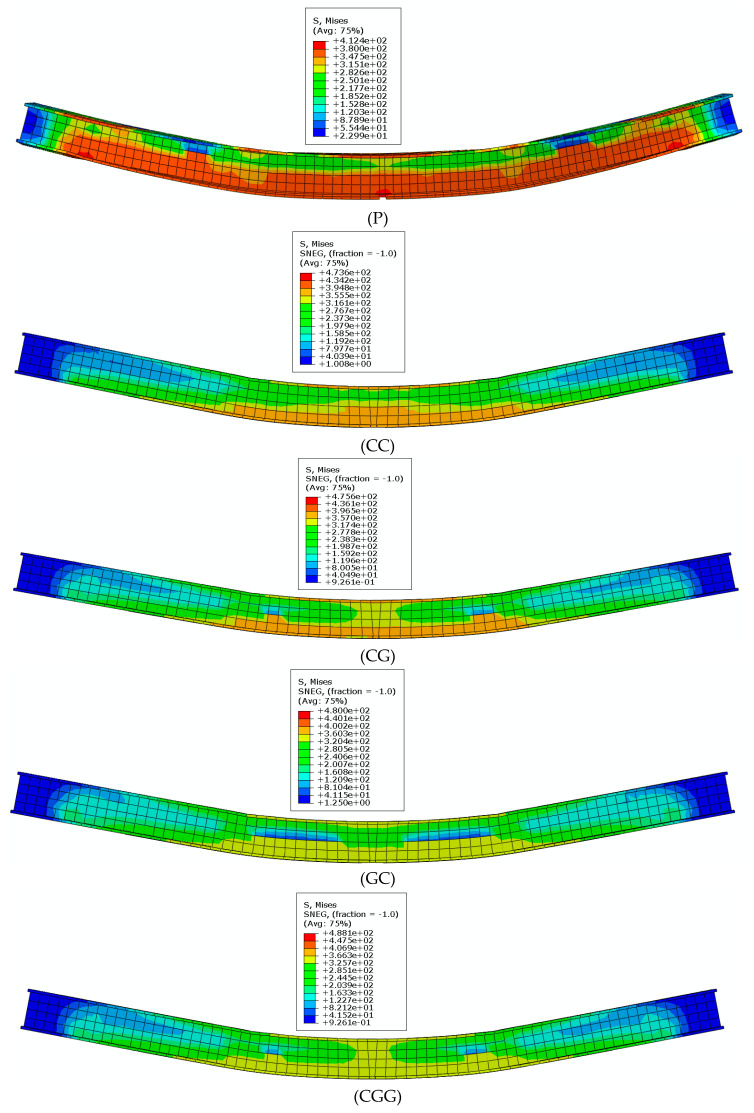

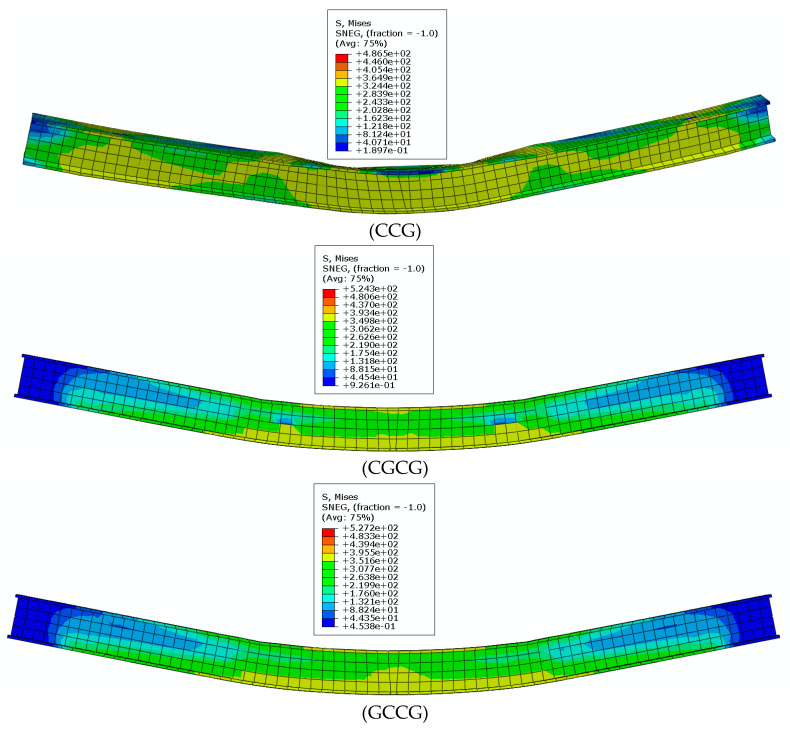
Stress nephogram of test pieces.

Through stress nephogram analysis, it was found that the stress concentration of the reinforced beam was distributed in the mid-span area of the lower half of the web and the bottom area of the beam. The overall stress distribution pattern is that the stress gradually increases from the upper part to the lower part of the steel beam, and decreases from the middle to both sides of the span. The highest stress appears in the lower half of the beam span. Comparing specimen P with each reinforced beam, the CFRP/GFRP cloth reinforcement effectively reduces the stress on the beam web and lower flange and improves the stress distribution. The stress cloud diagram of each specimen beam is shown in Figure 9.

### 4.3. Stress Process Analysis

**Figure 10:**
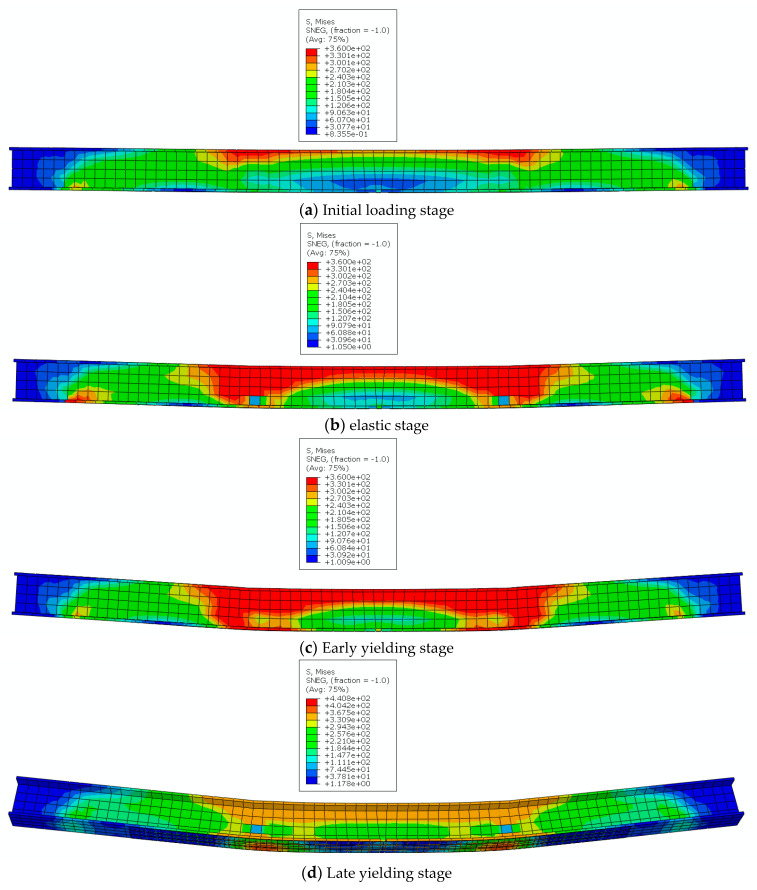

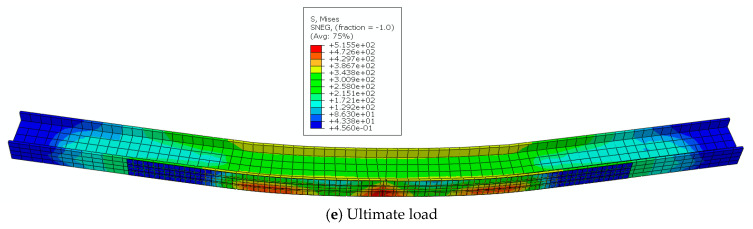
Stress nephogram of test piece CGCG at different times.

Taking the CGCG of a four-story reinforced beam as an example, compare the stress cloud maps at different times and analyze the stress development law. As shown in Figure 10.

When the reinforced beam is initially loaded, stress is generated at the upper flange in contact with the loading point, and the high-stress area is concentrated at the loading point and spreads around, resulting in significant stress between the two loading points. The steel beam web plays a role in resisting shear and bending moments, resisting stress generated by force deformation, while the blue areas at both ends of the steel beam are not involved in the work and are displayed in blue. At this point, the stress in the bottom span of the steel beam is relatively small, and the CFRP/GFRP fabric is not involved in the stress, resulting in a low utilization rate of CFRP/GFRP fabric. As shown in Figure 10a. At the initial stage of applying load, the reinforced beam is in the elastic stage, and the stress in the loading point area begins to transfer downwards from the loading point to the web. The high-stress area of the web has significantly expanded, indicating that the steel beam web further participates in the work of resisting bending deformation. The balance of stress in the mid-span area of the beam also generates corresponding support reactions at the support, resulting in an increase in stress values. At this point, there was no significant deformation of the reinforced beam as a whole, as shown in Figure 10b. As the load value increases, significant bending deformation is observed in the reinforced beam, and the high-stress area at the lower flange of the beam at the loading point further expands, as shown in Figure 10c. As the load value increases, the overall stress value of the steel beam increases, and the CFRP/GFRP cloth pasted at the bottom further plays a reinforcement role. The high stress is concentrated on the corresponding CFRP/GFRP cloth at the load application position, as shown in Figure 10d. As the load increases and the bending deformation of the reinforced beam increases, the CFRP/GFRP fabric is constantly subjected to stress, which is transmitted from both sides to the mid-span. The maximum stress area appears at the mid-span loading point, ultimately leading to peeling or fracture of the CFRP/GFRP fabric. The stress at the upper web of the beam decreases slightly due to elastic recovery, as shown in Figure 10e.

## 5. Conclusions

The experimental and numerical simulation research on the hybrid reinforcement of corroded I-shaped steel beams with CFRP/GFRP cloth mainly draws the following conclusions:

(1) Based on the experimental failure phenomena of 8 test beams, 2 failure modes were identified, namely the middle section torsion instability failure of the steel beam and the fracture and detachment of the CFRP/GFRP cloth from the steel beam.
(2) The overall strain value of the reinforced beam increases continuously with the increase of load. After the steel beam enters the yield stage, significant bending deformation occurs in the mid-span area, where the maximum tensile stress of CFRP/GFRP fabric is concentrated here. Therefore, the fracture and peel failure of CFRP/GFRP fabric also occur here.
(3) The number of CFRP/GFRP reinforcement layers is the most important factor affecting the reinforcement effect. Compared to the unreinforced beam, the yield load increase rate of the 2-layer CFRP/GFRP fabric is between 2.78% and 5.95%, and the ultimate load increase rate is between 7.23% and 8.65%. The reinforcement effect of 3-layer CFRP/GFRP fabric increase rates ranging from 12.31% to 13.58%, and ultimate load increase rates ranging from 12.88% to 15.28%.The effect of 4-layer CFRP/GFRP fabric has been significantly improved, with yield load increase rates ranging from 17.68% to 20.19%, and ultimate load increase rates ranging from 25.03% to 27.34%.
(4) When the number of CFRP reinforcement layers is the same, the number of GFRP reinforcement layers is directly proportional to the yield load and ultimate load of the reinforced beam. GFRP fabric effectively suppresses the plastic development of the tensile zone of the specimen and improves the flexural bearing capacity of the corroded I-shaped steel beam.

## Figures and Tables

**Table 1:** Test piece grouping. C refers to CFRP; G refers to GFRP.

Number	Interlayer Mixed Layer	Number	Interlayer Mixed Layer
P	0 layer CFRP/0 layer GFRP	CGG	1 layer CFRP/2 layers GFRP
CC	2-layer CFRP	CCG	2 layers of CFRP/1 layer of GFRP
CG	0 layer CFRP/0 layer GFRP	CGCG	1 layer CFRP/1 layer GFRP/1 layer CFRP/1 layer GFRP
GC	1 layer GFRP/1 layer CFRP	GCCG	1 layer GFRP/2 layers CFRP/1 layer GFRP

**Table 2:** Quality Comparison of Rust Damaged Steel Beams. C refers to CFRP; G refers to GFRP.

Number	Quality	Quality after Rusting	Mass Loss (%)	Number	Quality	Quality after Rusting	Mass Loss (%)
	(kg)	(kg)			(kg)	(kg)	
P	10.41	10.04	3.55%	CGG	10.42	9.97	4.32%
CC	10.44	9.98	4.41%	CCG	10.47	9.99	4.58%
CG	10.43	9.95	4.60%	CGCG	10.43	10.03	3.84%
GC	10.46	10.06	3.82%	GCCG	10.43	10.11	3.07%

**Table 3:** Mechanical property parameters of CFRP and GFRP materials.

FRP Type	Nominal Thickness (mm)	Performance Parameter	
		Project	Performance Parameter
Carbon fiber cloth	0.167	tensile strength (MPa)	3468
		Tensile modulus of elasticity (MPa)	2.31 × 10^5^
		tensile elongation at break (%)	1.74
Glass fiber cloth	0.167	tensile strength (MPa)	3356
		Tensile modulus of elasticity (MPa)	8.71 × 10^4^
		tensile elongation at break (%)	5.3

**Table 5:** Comparison of failure modes of test pieces. C refers to CFRP; G refers to GFRP.

Test Piece Number	Destruction Mode
P	During the loading process of the steel beam, the upper and lower flanges of the span buckled and deformed forward, resulting in the instability of the specimen and the inability to load.
CC	The CFRP/GFRP fabric broke at the anchorage and detached from the steel beam.
CG	Part of the fibers in the middle of the CFRP/GFRP fabric are broken, and the CFRP/GFRP fabric is detached from the steel beam.
GC	The CFRP/GFRP fabric on one side of the steel beam is broken, the CFRP fabric is detached from the GFRP fabric, and the inner layer of GFRP fabric is not completely pulled apart.
CGG	The middle part of the CFRP/GFRP fabric is detached from the steel beam, and there is a peeling phenomenon between one side of the U-shaped hoop and the web plate.
CCG	The CFRP/GFRP fabric breaks and detaches from the steel beam, causing a sudden increase in the degree of buckling and bending at the upper flange of the steel beam span, resulting in the instability of the steel beam.
CGCG	The outer GFRP fiber at one edge experienced partial fiber breakage and cracking, with some CFRP/GFRP cloth detached from the steel beam and the specimen damaged.
GCCG	Multiple fiber fractures and detachment occurred, and the middle of the CFRP/GFRP fabric detached from the steel beam.

**Table 6:** Comparison of load-bearing capacity of specimens with different GFRP cloth reinforcement layers. C refers to CFRP; G refers to GFRP.

Test Piece Number	Yield Load (kN)	Yield Load Increase Rate (%)	Ultimate Load (kN)	Ultimate Load Increase Rate (%)
CC	37.32	/	45.97	/
CCG	40.78	9.27	48.39	5.26
CGCG	43.64	16.93	53.60	16.60
GCCG	42.95	15.09	54.12	17.73

**Table 7:** Comparison of load-bearing capacity of specimens reinforced with GFRP fabric instead of CFRP fabric.

Test Piece Number	Yield Load (kN)	Yield Load Increase Rate (%)	Ultimate Load (kN)	Ultimate Load Increase Rate (%)
CC	37.32	/	45.97	/
CG	38.47	3.08	46.46	1.07
GC	38.06	1.98	46.58	1.33
CCG	40.78	/	48.39	/
CGG	41.24	1.13	49.42	2.13

**Table 8:** Analysis of the bearing capacity of I-shaped steel beams with corroded defects reinforced by CFRP/GFRP hybrid reinforcement.

Test Piece Number	Yield Load (kN)	Yield Load Increase Rate (%)	Ultimate Load (kN)	Ultimate Load Increase Rate (%)
CC	37.32	/	45.97	/
CG	38.47	3.08	46.46	1.07
GC	38.06	1.98	46.58	1.33
CCG	40.78	/	48.39	/
CGG	41.24	1.13	49.42	2.13

## Data Availability

Not applicable.

## Nomenclature

**Symbol**	**Name**
E	Elastic modulus
Nu	Poisson’s ratio
G	shear modulus
